# Stapled Transanal Rectal Resection (Starr) in the Treatment of Obstructed Defecation: A Systematic Review

**DOI:** 10.3389/fsurg.2022.790287

**Published:** 2022-02-14

**Authors:** Lorenzo Ripamonti, Angelo Guttadauro, Giulia Lo Bianco, Maria Rennis, Matteo Maternini, Gerardo Cioffi, Marco Chiarelli, Matilde De Simone, Ugo Cioffi, Francesco Gabrielli

**Affiliations:** ^1^Department of Medicine and Surgery, University of Milano-Bicocca, Milan, Italy; ^2^General Surgery Department, Istituti Clinici Zucchi Monza, Monza, Italy; ^3^Department of Sciences and Technologies, University of Sannio RCOST, Benevento, Italy; ^4^Department of Surgery, Ospedale Alessandro Manzoni, ASST Lecco, Lecco, Italy; ^5^Department of Surgery, University of Milan, Milan, Italy

**Keywords:** obstructed defecation syndrome, stapled trans-anal rectal resection, QOL, international guidelines, outcomes, surgical procedure

## Abstract

Obstructed defecation syndrome (ODS) is a form of constipation that influences the quality of life in most patients and is an important health care issue. In 2004 Longo introduced a minimal invasive trans-anal approach known as Stapled Trans-Anal Rectal Resection (STARR) in order to correct mechanical disorders such as rectocele or rectal intussusception, two conditions present in more than 90% of patients with ODS. Considering the lack of a common view around ODS and STARR procedure. the aim of our study is to review the literature about preoperative assessment, operative features and outcomes of the STARR technique for the treatment of ODS. We performed a systematic search of literature, between January 2008 and December 2020 and 24 studies were included in this review. The total number of patients treated with STARR procedure was 4,464. In conclusion STARR surgical procedure has been proven to be safe and effective in treating symptoms of ODS and improving patients Quality of Life (QoL) and should be taken in consideration in the context of a holistic and multi modal approach to this complex condition. International guidelines are needed in order to optimize the diagnostic and therapeutic process and to improve outcomes.

## Introduction

Twenty-six percent of people in Europe suffer from chronic constipation. This condition influences the quality of life in most patients (1) and is an important health care issue (2).

Obstructed defecation syndrome (ODS) is a form of constipation characterized by impaired defecation that consists in fragmented stool, need for straining at defecation, sense of incomplete evacuation, tenesmus, urgency, pelvic heaviness and need for self-digitation (3), use of digital assistance or enemas, bleeding and pain (4, 5). ODS is most commonly found in middle aged women (6).

From an etiological standpoint ODS be caused by functional or mechanical disorders (7). Sometimes they coexist or can be one the consequence of the other. Functional abnormalities are more difficult to diagnose and frequently need a complex therapeutic approach involving psychologists, neurologists, physiatrists and sometimes surgeons (3). Surgery alone is not a solution for these patients as demonstrated by Vermeulen et al. (8).

On the other side we have mechanical disorders such as rectocele or rectal intussusception, two conditions present in more than 90% of patients with ODS (3, 9). Several therapeutic approaches have been described for these organic disorders including trans-anal, transvaginal, trans-perineal and abdominal approaches (10). In 2004 Longo introduced a minimal invasive trans-anal approach known as Stapled Trans-Anal Rectal Resection (STARR) (11). STARR procedure consists of an endorectal resection of the distal rectum using a stapler (12–14).

There are controversial opinions around efficacy and side effects of this procedure. For this reason, on 2006, the European STARR registry was founded with the aim to register all STARR procedures and outcomes through a collaboration between surgical societies from Italy, Germany, UK, North European Countries and France (15). The first results were published on 2009 (16) showing good outcomes in term of quality of life and complications rates. On the other side, some studies have shown important complications such as pelvic sepsis, fistulas, fecal urgency, post-operative bleeding (17).

There is no agreement on risks and benefits about the STARR procedure for ODS. The aim of our study is to show the state of the art and carry out a review of the most important literature published on preoperative assessment and operative features of STARR procedure for ODS. The main purpose is to highlight clinical outcomes for treatment of ODS using STARR in particular recurrence rate, symptoms reduction and patients' quality of life.

## Methods

According to the Preferred Reporting items for Systematic Reviews and Meta-Analyses (PRISMA) statement (18), we performed a search of literature, between January 2008 and December 2020 (last search on march 2021). LR and MR conducted the search employing the PubMed (Medline) and Scopus database using mesh and free text words and selecting original works concerning the application of STARR in patients with ODS. Our search was limited to human studies, available in full text, published in English language as original contributions. Case reports were discarded (Table 1). Some works were subsequently discarded after collegial discussion among LR, MR, GB, and AG because they were considered not strictly related to the topic taken into consideration. The quality of the studies was evaluated by examining three factors: patient selection, compatibility with the purpose of the research and evaluation of the result.

**Table 1:** PICOS table.

**Participants**	**Human, > 18 years old, diagnosis of obstructed defecation with indication to surgery**
Interventions	STARR technique using PPH01, PPH03, TST, STR10, CCS30, STR5G
Comparisons	-
Outcomes	Preoperative assessment, operative features and post-operative complications and long-term outcomes of the STARR technique for the treatment of ODS
Study design	Retrospective, prospective and randomized control trials available in full text and published in English language. Case reports were excluded

**Figure 1:**
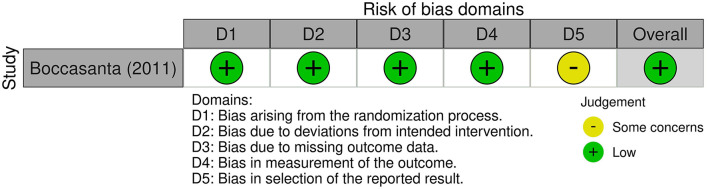
Risk of bias according to RoB 2 tool for RCT.

**Figure 2:**
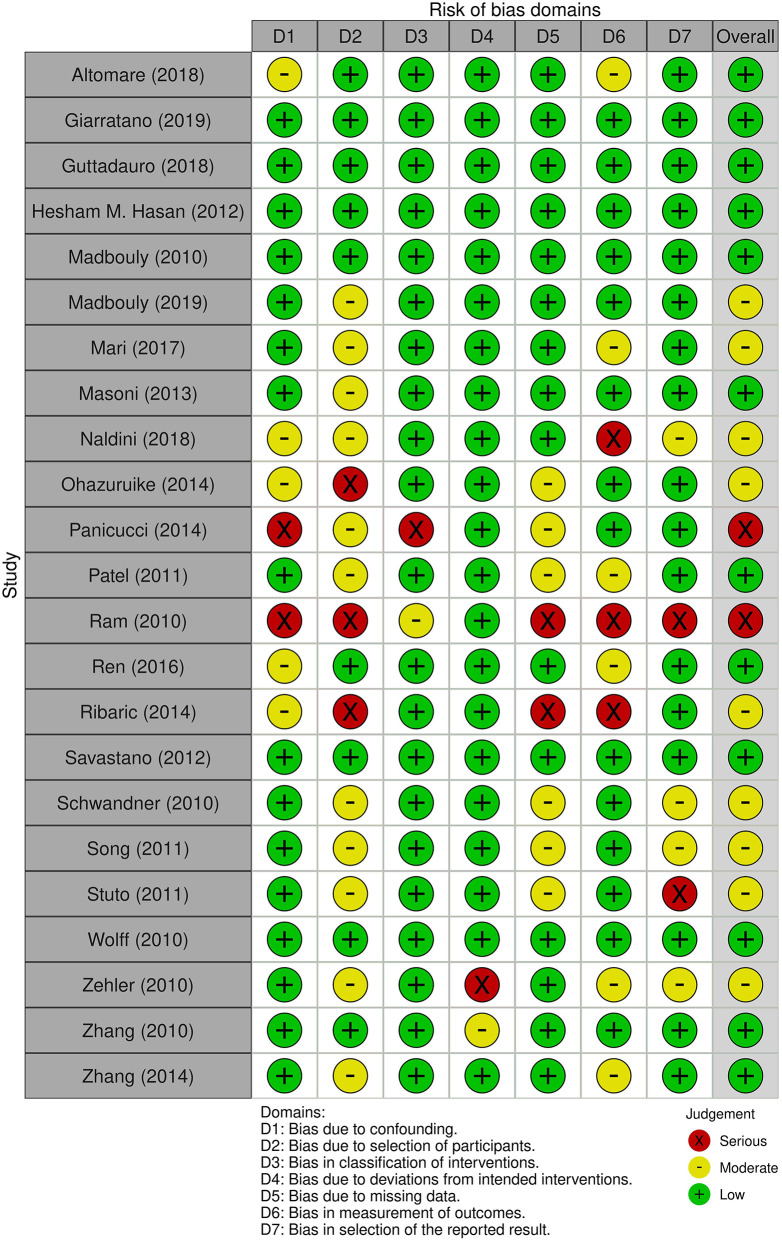
Risk of bias according to ROBINS-I tool.

The risk of bias assessment of the included studies was performed by LR and MR using RoB 2 tool for randomized-controlled trials (RCT) and ROBINS-I tool for the other studies (Figures 1, 2).

**Figure 3:**
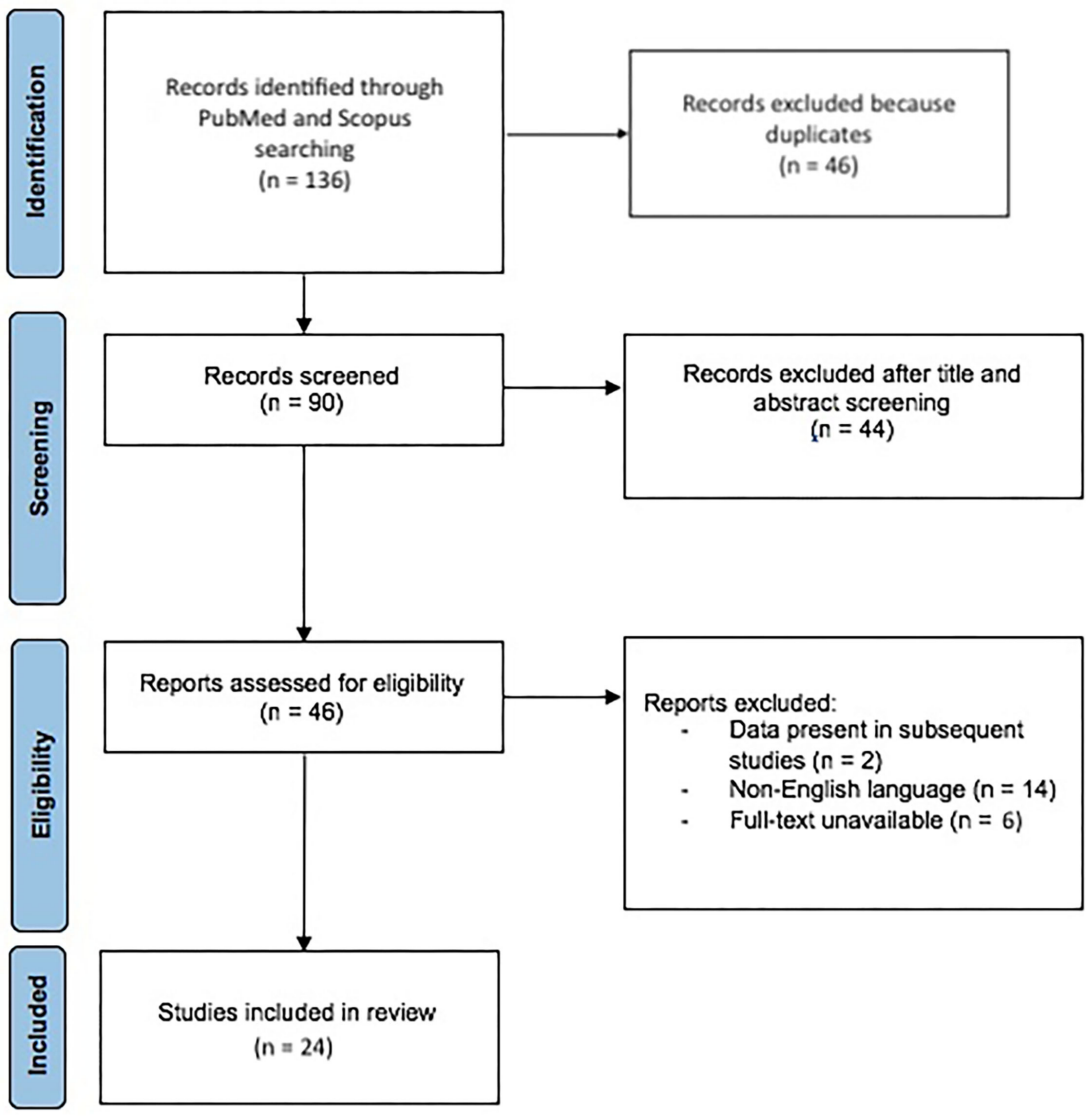
PRISMA 2020 flow diagram for study selection.

We conducted our search indicating the following terms to be present in in title/abstract using the PubMed (Medline) advanced search function: Stapled Trans-Anal Rectal Resection or STARR and obstructed defecation or ODS. A total of 136 results was found, 46 of them were excluded because duplicates and 44 studies were excluded after title and abstract screening. Among 46 articles accessed for eligibility, 20 were excluded because non-English language (*n* = 14), full-text unavailable (*n* = 6) or data present in subsequent studies (*n* = 2). The remaining 24 studies were included in this review (Figure 3).

## Results

### Patients Characteristics

We analyzed twenty-four studies about STARR technique divided in retrospective, prospective and randomized control trials (9, 10, 15, 19–39) (Table 2). The total number of patients treated with STARR procedure was 4,464. There was a significant difference concerning gender as 88 per cent of patients were found to be women. Patients' average age was 61 years. The majority of patients had experienced conservative treatment before surgery. In total 1,272 (28%) patients had a history of previous pelvic surgery.

**Table 2:** Studies.

**Author**	**Kind of study**	**N patients**	**F/M**	**Age**	**Preoperative conservative treatment**	**Previous pelvic surgery**
Altomare et al. (19)	Retrospective	21	21/0	58 (32–76)		
Boccasanta et al. (20)	RCT	50	50/0	54.8 (27–77)	-	N
		50	50/0	57.1 (31–74)	-	
Giarratano et al. (21)		260	260/0	54 (20–78)	Y	Y
Guttadauro et al. (22)	Retrospective	450	387/63	56 (28–77)	Y	-
Hasan and Hasan (23)	Prospective	40	40/0	45.7 (30–63)	Y	Y
Madbouly et al. (24)	Retrospective	46	30/16	48.4 (29–68)	Y	-
Madbouly et al. (25)	Randomized	56	38/18	75 (70–85)	Y	Y
Mari et al. (26)	Retrospective	96	93/3	55 (33–82)	Y	-
Masoni et al. (27)		187	182/5	56 (33–85)	Y	Y
Naldini et al. (28)	Retrospective	45	45/0	50.1 (24–79)		-
Ohazuruike et al. (29)	Retrospective	23	19/4	52.6	Y	Y
Panicucci et al. (30)	Prospective	54	52/2	54.25 (28–77)	Y	-
Patel et al. (31)	Retrospective	37	37/0	52.9 (31–74)	Y	Y
Ram et al. (32)		30	29/1	67.1 (50–75)	-	-
Ren et al. (33)	Retrospective	50	43/7	53 (22–82)	Y	-
Ribaric et al. (15)	Prospective	100	98/2	60 (27–82)	Y	Y
Savastano et al. (34)	Prospective	32	32/0	54	Y	-
		32	32/0	60.5		
Schwandner et al. (9)	Prospective	379	296/83	57.8	Y	Y
Song et al. (35)	Retrospective	58	50/8	54 (19–85)	Y	Y
Stuto et al. (36)	Prospective	2,171	1,653/358	56.28 (20–96)	-	-
Wolff et al. (37)	Prospective	52	52/0	64 (20–87)	Y	Y
Zehler et al. (38)	Prospective	20	19/1	60.5 (45.3–78.6)	Y	-
Zhang et al. (10)	Retrospective	50	50/0	53.7 (30–70)	Y	Y
Zhang et al. (39)	Prospective	75	75/0	54.3 (29–75)	Y	Y

*RCT, randomized controlled trial; Y, yes; N, not; F, female; M, male*.

### Preoperative Assessment

#### Diagnosis and Preoperative Evaluation

All patients underwent a preoperative assessment before going to surgery with different tests to evaluate the pelvic floor function and to exclude disorders that could contraindicate surgery (9, 10, 15, 19–39) (Table 3). The almost totality of patients, in all of the analyzed studies, performed a dynamic defecography (*n* = 4,512, 99%) and an endoscopic study such as colonoscopy or proctoscopy (*n* = 4,276, 94%) and completed the assessment with an anorectal manometry (*n* = 4,029, 88%). Only 37 (0.9%) patients in the retrospective study of Patel et al. (31) performed balloon expulsion test (BET) to exclude pelvic floor dyssynergia. Approximately 50% of patients (*n* = 2,297) had a trans-anal ultrasound and 846 (18%) patients underwent MRI defecography.

**Table 3:** Preoperative evaluation.

**Author**	**Dynamic**	**Anorectal**	**Proctoscopy/**	**Electro-**	**Balloon**	**Rectal**	**Transanal**	**MRI**	**Transit**
	**defecography**	**manometry**	**colonoscopy**	**miography**	**expulsion**	**sensation**	**US**	**defecography**	**time**
Altomare et al. (19)	Y	Y	Y	N	N	N	N	N	N
Boccasanta et al. (20)	Y	Y	Y						
									
Giarratano et al. (21)	Y	Y	Y	N	N	N	N	N	N
Guttadauro et al. (22)	Y	Y	Y	N	N	N	N	N	N
Hasan and Hasan (23)	Y	N	Y	N	N	N	N	N	N
Madbouly et al. (24)	Y	Y	Y	N	N	N	N	N	N
Madbouly et al. (25)	Y	N	N	N	N	N	N	N	N
Mari et al. (26)	Y	Y	Y	N	N	N	N	Y	N
Masoni et al. (27)	Y	Y	N	N	N	N	N	Y	N
Naldini et al. (28)									
Ohazuruike et al. (29)	Y	Y	Y	N	N	N	N	N	N
Panicucci et al. (30)	Y	Y	Y	N	N	N	Y	N	N
Patel et al. (31)	Y	Y	Y	Y	Y	Y	N	N	
Ram et al. (32)	Y	Y	Y	Y	N	N	N	N	N
Ren et al. (33)	Y	N	Y	N	N	N	N	N	N
Ribaric et al. (15)	Y	N	Y	N	N	N	N	Y	N
Savastano et al. (34)	Y	Y	Y					Y	
									
Schwandner et al. (9)	Y		Y					Y	
Song et al. (35)	Y	Y	Y	Y	N	N	N	N	Y
Stuto et al. (36)	Y	Y	N	N	N	N	Y	N	N
Wolff et al. (37)	N	Y	Y	N	N	N	Y	Y	N
Zehler et al. (38)	Y	Y	Y	N	N	N	Y	N	
Zhang et al. (10)	Y	Y	Y	N	N	N	N	N	Y
Zhang et al. (39)	Y	Y	Y	N	N	N	N	N	Y

*Y, yes; N, not; US, ultrasound; MRI, magnetic resonance imaging*.

#### Scoring Systems

As shown in Table 4 (9, 10, 15, 19–39) 85% of patients (*n* = 3,876) were evaluated using Obstructed Defecation Syndrome Score (ODS score) obtaining a mean result of 16 out of 36 before surgery. Approximately 22% of patients (*n* = 1,018) were also scored according to the Wexner Constipation Score before going to surgery obtaining a mean result of 14.5 (in a range between 0 and 30). Sixty percent of patients (*n* = 2,720) were presented with a Symptoms Severity Score questionnaire (SS score) before surgery reaching a mean score of 12 out of 19. Some authors also analyzed the impact of surgery on patients' quality of life: 63% of patients (*n* = 2,870) answered to the Patient Assessment of Constipation Quality of life questionnaire (PAC-QoL) before going to surgery obtaining a mean result of 50 (range 0–112).

**Table 4:** Score for preoperative evaluation.

**Author**	**ODS score**	**Wexner constipation score**	**SS score**	**PAC-QoL**	**Enema/ bowel prep**
Altomare et al. (19)	16.00				
Boccasanta et al. (20)	20.60				Y
	20.88				
Giarratano et al. (21)		19.00			Y
Guttadauro et al. (22)	14.10				Y
Hasan and Hasan (23)	14.20				Y
Madbouly et al. (24)	11.56			52.60	
Madbouly et al. (25)	17.40	11.10		55.10	
Mari et al. (26)		15.80			Y
Masoni et al. (27)		15.80			Y
Naldini et al. (28)	17.26				
Ohazuruike et al. (29)	18.20	17.00			Y
Panicucci et al. (30)	21.38	19.49			Y
Patel et al. (31)		11.10			Y
Ram et al. (32)	17.10				Y
Ren et al. (33)		13.96			Y
Ribaric et al. (15)	15.65				Y
Savastano et al. (34)	13.00		12.00		-
	15.00		14.00		
Schwandner et al. (9)	11.14		13.00		-
Song et al. (35)		17.60			Y
Stuto et al. (36)	16.70		15.60	51	Y
Wolff et al. (37)	16.00	12.50			Y
Zehler et al. (38)	8.00	4.00	5.00		Y
Zhang et al. (10)	17.54	15.58	12.22	47.78	Y
Zhang et al. (39)	18.39	15.57	13.69	44.45	Y

*ODS, Obstructed Defecation Syndrome; SS, Symptoms Severity; PAC-QoL, Patient Assessment of Constipation Quality of life questionnaire*.

#### Preoperative Management

The preoperative setting before STARR is similar in most centers (Table 5) (9, 10, 15, 19–39). Patient is usually prescribed a preoperative enema while only in rare cases, bowel preparation is administered orally.

**Table 5:** Surgical feature.

**Author**	**Antibiotic profilaxis**	**Anesthesia**	**Stapler**	**Time (min)**	**LOS (d)**
Altomare et al. (19)	-	-	PPH01	-	-
Boccasanta et al. (20)	Cefotaxime + metronizadole	Spinal	PPH01	42.4	3.2
			CCS30	52.2	3.5
Giarratano et al. (21)	Cefotaxime	General/spinal	PPH01/PPH03	42.0	3
Guttadauro et al. (22)	Cefotaxime + metronidazole	Spinal	PPH01	30.2	1
Hasan and Hasan (23)	Y	General/spinal	PPH01	35.0	1.7
Madbouly et al. (24)	-	General	PPH01	48.4	1
Madbouly et al. (25)	-			45.4	1
Mari et al. (26)	Metronidazole + ciprofloxacin/cefotaxime	General/spinal	CCS30	-	-
Masoni et al. (27)	Metronidazole + ciprofloxacin or metronidazole + cefotaxime	General	CCS30	48.0	3
Naldini et al. (28)	-	General/spinal	TST	30.9	2.6
Ohazuruike et al. (29)	Metronidazole + cefamezin	General	PPH01	28.0	2
Panicucci et al. (30)	Metronidazole + cefamezin	General/spinal	PPH01/CCS30	-	-
Patel et al. (31)	Y	General	STR10	-	1
Ram et al. (32)	Metronidazole + ceftriaxone	General/spinal	PPH01	40.0	2
Ren et al. (33)	Y	General/spinal	TST	21.0	5
Ribaric et al. (15)	Y	General/spinal	CCS30	43.8	4.46
Savastano et al. (34)	Y	Spinal	PPH01	28.0	2
			CCS30	43.0	4
Schwandner et al. (9)	-	General/spinal	PPH01	40.0	5.5
Song et al. (35)	Metronidazole + cefotaxime	Spinal	PPH01	35.1	3.91
Stuto et al. (36)	Y	General/spinal	PPH01/CCS30	95.0	3.54
Wolff et al. (37)	Metronidazole + cefamandole	General/spinal	STR5G	45.0	5
Zehler et al. (38)	Y	General/spinal	PPH01	-	8
Zhang et al. (10)	Y	Spinal	PPH01	28.0	-
Zhang et al. (39)	Y	Spinal	PPH01	30.0	5

*LOS, lenght of stay; min, minutes; d, days; Y, yes*.

The bacterial flora of the anal canal is composed of aerobic and anaerobic bacteria (40) for this reason antibiotic prophylaxis involves the use of antibiotics capable of acting on the entire bacterial spectrum. Metronidazole combined with a cephalosporin or ciprofloxacin is commonly administered as antibiotic prophylaxis, immediately after the induction of anesthesia. In most articles, either general or spinal anesthesia is practiced.

#### Surgical Technique and Devices

As described for the first time by Longo (11) the STARR technique consists in a full thickness resection of the anterior and posterior rectal wall (including mucosa, submucosa, and rectal muscle wall) firing two circular staplers. The PPH01 stapler (Ethicon Endo-Surgery Inc.) was the first to be employed and nowadays it is still the most used. A new version of this circular stapler has been developed, the PPH03 (Ethicon Endo-Surgery Inc.), featuring a minor height of the closed staples, e but only one study using this stapler was found (28). It evaluated outcomes after using the TST (Touchstone International Medical Science Co.).

In 2008 Renzi et al. (41) proposed the Transtar a revised version of the STARR technique using a new dedicated device, a rechargeable CCS-30 Contour Transtar stapler kit (Ethicon Endo-Surgery Inc.). Subsequently other devices for the Transtar were produced like STR10 Transtar (Ethicon Endo-Surgery Inc.) and the Contour Transtar TM-STR5G (Ethicon Endo-Surgery Inc.).

#### Short Term Outcomes

Median LOS was 3.65 days while median operative time was 64.33 min.

Possible postoperative complications include: bleeding which can present itself early (early rectal bleeding) or be delayed and lead to the formation of a stable pelvic hematoma, stapled line complications (bleeding, infection, partial dehiscence, pelvic sepsis due to sub-peritoneal perforation, anastomotic leakage, granuloma), vaginal tears, the development of a recto vaginal fistula, fecal urgency, postoperative pain, stricture/stenosis.

Following the trend of most authors, we focused on pain/tenesmus, urinary retention, rectal bleeding, pelvic hematoma, anastomotic dehiscence, granuloma, rectovaginal fistula, urgency. Relative reported frequencies vary between different authors and are reported in Table 6 (9, 10, 15, 19–39). Urgency is the most frequent complication in the immediate post-surgical phase with reported rates up to 47.8%. This symptom however, tends to decrease over time as shown in the analysis below. The second most frequent short-term complication is pain/tenesmus (ranging between 0.4 and 24%). Urinary retention occurs between 1.1 and 9.6% of cases and bleeding reported rates vary between 0.5 and 12.5%. Anastomotic dehiscence is the most feared complication and rates range between 0.4 and 7.1%. Vaginal tears and stapled line granulomas are seldom reported.

**Table 6:** Postoperative complications.

**Author**	**Pain/tenesmus**	**Urinary**	**Rectal**	**Pelvic**	**Anastomotic**	**Granuloma**	**Rectovaginal**	**Urgency**
	**(%)**	**retention (%)**	**bleeding (%)**	**hematoma (%)**	**dehiscence (%)**	**(%)**	**fistula (%)**	**(%)**
Altomare et al. (19)	1 (0.1)							2 (0.3)
Boccasanta et al. (20)			2 (4)				0	17 (34)
			0				0	7 (14)
Giarratano et al. (21)	10 (4)	18 (6.8)	12 (4.5)	1 (0.4)	1 (0.4)		1 (0.4)	27 (10.3)
Guttadauro et al. (22)	0	35 (7.8)	13 (2.9)	5 (1.1)	19 (4.2)	0		125 (17.8)
Hasan and Hasan (23)	4 (10)	2 (5)	4 (10)	0	0	0	0	16 (40)
Madbouly et al. (24)	1 (2.2)	1 (2.2)	0	0	0		0	
Madbouly et al. (25)	3 (2.4)		2 (1.78)				0	8 (7.14)
Mari et al. (26)	20 (20.8)							8 (8.3)
Masoni et al. (27)				4 (2.1)			1 (0.5)	12 (6.4)
Naldini et al. (28)	11(24.4)			1 (2.2)				12 (26.6)
Ohazuruike et al. (29)			1 (4)					11 (47.8)
Panicucci et al. (30)								
Patel et al. (31)	5 (13.5)	1 (2.7)	6 (16.2)	0	0	2 (5.4)	0	2 (5.4)
Ram et al. (32)	3(10)		0					
Ren et al. (33)	12 (24)		1 (0.5)					5 (10)
Ribaric et al. (15)	1(1)	2(2)	5(5)					
Savastano et al. (34)			4 (12.5)					29 (29.8)
				1(3.12)	2 (6.25)			6 (18.7)
Schwandner et al. (9)	2 (0.5)	4 (1.1)	11 (2.9)		27 (7.1)	4 (1.1)		6 (1.6)
Song et al. (35)	1 (1.7)		7 (12)					11 (18.9)
Stuto et al. (36)		209 (9.63)	79 (3.6)		74 (3.4)		1 (0.005)	567 (26.1)
Wolff et al. (37)	2 (3.07)	1 (1.6)	2 (2.9)					
Zehler et al. (38)			2 (2.9)			1 (1.4)	0	
Zhang et al. (10)	2 (4)	2 (4)	4 (8)	0	0	0	0	21 (42)
Zhang et al. (39)		4 (4.65)	6 (6.9)					30 (35)

#### Long Term Outcomes

Long-term effects of surgery can be measured in terms of recurrence rate, symptoms reduction and patients' quality of life.

Authors employed different scoring systems to assess obstructed defecation syndrome including: obstructed defecation syndrome and modified obstructed-defecation syndrome questionnaire (ODS and MODS), Wexner incontinence score, Symptom Severity Score (SS), Agachan-Wexner constipation score, CGS continence grading scale. The impact of surgery on everyday life was measured with the constipation quality of life (PAC-QOL) and Euro Quality of Life-5 Dimension (EQ-5D) score. In some series, patients underwent postoperative anorectal manometry and/or defecography.

In the Table 7 we focused on the most frequently cited long term outcomes. More specifically, we analyzed the reported rate of persistent or recurrent constipation, urgency at 3 months, 6 months, 1 year and 5 years from surgery, ODS score at 1 year, 3 years and 5 years from surgery, Wexner Score at 1 year, 3 years and 5 years from surgery, SS score at 1 year from surgery and PAC-QoL at 1 year and 3 years from surgery.

**Table 7:** Long term outcomes.

**Author**	**Persistent constipation /recurrence (%)**	**Urgency 3m (%)**	**Urgency 6m (%)**	**Urgency 1y (%)**	**Urgency 5y (%)**	**ODS score 1y**	**ODS score 3y**	**ODS score 5y**	**Wexner score 6m**	**Wexner Score 1y**	**Wexner score 3y**	**Wexner score 5y**	**SS Score 1y**	**PAC-QoL 1y**	**PAC-QoL 3y**
Altomare et al. (19)						12									
Boccasanta et al. (20)	6 (12)						3.52								
	0						3.14								
Giarratano et al. (21)	10 (4)								9						
Guttadauro et al. (22)	0	12 (5.3)	0			3.1	4.3	6.4		-		-			
Hasan and Hasan (23)	4 (10)	4 (10)	2 (5)	1 (2.5)		2.3									
Madbouly et al. (24)	3 (6.5)					2.2	3.7							30.3	40.9
Madbouly et al. (25)	11 (24)					6.7	10.2							14.7	20.2
Mari et al. (26)		3 (3.1)	1 (1)		1 (1)				5.2			7.4			
Masoni et al. (27)									5.2						
Naldini et al. (28)	3 (6.7)		4 (8.9)	2 (4.4)		4.74									
Ohazuruike et al. (29)						5.5			4.7						
Panicucci et al. (30)	3			4 (7.4)		5.47				6.14					
Patel et al. (31)	3 (8.1)									4.6					
Ram et al. (32)	4														
Ren et al. (33)									7.28	8.10					
Ribaric et al. (15)						5.52								0.95	
Savastano et al. (34)		29 (29.8)	23 (23.7)			1							1		
		6 (18.7)	6 (18.7)			1							1		
Schwandner et al. (9)						6.45							6.59	0.63	
Song et al. (35)	-									9.6					
Stuto et al. (36)			246 (11.3)	99 (4.56)		5				0.7			2.6	22	
Wolff et al. (37)		7(10.7)	4(6)	0		5			6						
Zehler et al. (38)	-					3		3				2	3		
Zhang et al. (10)	1 (2)	5 (10)	3 (6)	1 (2)		5.92				5.68			4.52	8.14	
Zhang et al. (39)	8 (10.7)			1 (1.3)		7.49	8.55			5.99	7.07		4.59	7	13.21

*ODS, Obstructed Defecation Syndrome; SS, Symptoms Severity; PAC-QoL, Patient Assessment of Constipation Quality of life questionnaire; m, months; y, years*.

Persistent and recurrent constipation are reported with a variable frequency ranging between 1 (1) and 24% (25) of patients according to different authors.

Excepting the study by Savastano (34), the reported postoperative urgency rates range between 3 and 10% and gradually decreasing over the years. At 5 years, Mari et al. (26) reports a 1% rate of urgency.

The median ODS score 1 year after surgery was 4.7 (mean 4.4, range 1–12) with a decrease over time (median 4 ranging between 3.14 and 10.2 at 3 years). Only two authors reported ODS score at five years after surgery (22, 38). Median Wexner score at 6 months and 1 year was 5.6 and 5.9 (mean 6.23, ranging between 4.7, 9, and 5.83 ranging between 0.7 and 9.6 respectively) with few data about its trend over time after the first year. Median SS score was 3 (mean 3.32 ranging between 1 and 6.59). Many authors measured the impact of surgery on patients' quality of life expressed in PAC-QoL that ranged between 0.63 and 30.3 1 year after surgery and between 13.21 and 40.9 3 years after surgery (median 8.14, mean 11.4 and median 20.2 and mean 24.7 respectively).

## Discussion

Obstructed defecation syndrome is a relatively frequent disorder with an important impact on the quality of life of patients that are usually women of working age (1). This disease has a complex etiology and often does not depend on anatomical conditions alone but also on functional abnormalities. Surgery can solve ODS related to anatomical abnormalities; at most if it is associated to medical support as psyllum fiber (42). Therefore, surgery should not be the first or the only therapeutic strategy. A multimodal approach is recommended (43). Patients' preoperatory evaluation should include a dynamic defecography (44) and an endoscopic study such as colonoscopy or proctoscopy. An anorectal manometry (40) completes the assessment.

The employ of clinical scores allows an accurate stratification of patients. The ODS score, ideated by Longo (11) is the most commonly employed and has shown a good correlation with ODS. Symptom Severity score (SS) evaluates 9 items on a maximum range of 36. Wexner score stratifies incontinence on the base of 5 including incontinence to liquid, solid, gas, necessity to wear pads and patient alteration. The impact of this condition on everyday life is measured with the constipation quality of life score (PAC-QOL).

Patients are operated on either under general or spinal anesthesia. Preoperative preparation includes the administration of an enema and antibiotic prophylaxis capable of acting on the entire bacterial spectrum. The most common employed antibiotic drugs are metronidazole combined with a cephalosporin or ciprofloxacin.

The surgical technique consists in a full thickness resection of the anterior and posterior rectal wall (including mucosa, submucosa, and rectal muscle wall) firing two circular staplers. The most commonly employed device is the PPH01 stapler. The introduction of new high-volume staplers like CPH34HV, CPH36 or TST36, could improve outcomes increasing the volume of prolapse resected in rectocele and rectal intussusception (45–47) allowing to treat major prolapses that have no indication to be treated with traditional staplers. Furthermore, the use of high-volume staplers could allow to use only one stapler (One Starr) with the same results as the starr made with 2 staplers, but more studies are needed.

Urgency is the most commonly described postoperative complication in the immediate post-surgical phase but tends to decrease over time. Pain, tenesmus, urinary retention and bleeding are reported with variable frequencies (48). Anastomotic dehiscence is the most feared but fortunately rare complication (49). Vaginal tears and stapled line granuloma are seldom reported.

Stypsis recurrence or persistence is reported between to 24% of cases according to different authors. Resistance is reported occurs 1 to 24% of cases according to different authors. ODS, Wexner and SS score decrease after the procedure show that the procedure is effective on ODS symptoms but there is a lack of data on long term follow up (longer than 1 or 3 years after the procedure). PAC-QoL score is often employed and shows an effect of surgery in ameliorating patient life in variable measure comparing different series. Results variability might depend on study population heterogeneity but also on the complex nature of this condition. Surgery alone is effective in the correction of rectocele and rectal internal mucosal prolapse, which are often present, but other functional and organic issues are often present and should be addressed in a well-coordinated multi modal approach. There is a lack of data on long-term effects which should be addressed in further studies.

The strength of our study is the inclusion of a high number of patients treated with STARR procedure despite the limitation due the use of a single database for data search. It would be useful to perform wider search using more databases and making a meta-analysis such made by Van Geluwe et al. (50).

This review highlights the effectiveness of STARR as ODS treatment but emphasizes the need to standardize the diagnostic process, the choice of the preoperative setting and the device, and the methodology of the entity assessment of symptoms and outcomes.

## Conclusion

STARR surgical procedure has been proven to be safe and effective in treating symptoms of ODS and improving patients QoL and should be taken in consideration in the context of a holistic and multi modal approach to this complex condition. International guidelines are needed in order to optimize the diagnostic and therapeutic process and to improve outcomes.

## Data Availability Statement

The original contributions presented in the study are included in the article/supplementary material, further inquiries can be directed to the corresponding author/s.

## Author Contributions

All authors listed have made a substantial, direct, and intellectual contribution to the work and approved it for publication.

## Conflict of Interest

The authors declare that the research was conducted in the absence of any commercial or financial relationships that could be construed as a potential conflict of interest.

## Publisher's Note

All claims expressed in this article are solely those of the authors and do not necessarily represent those of their affiliated organizations, or those of the publisher, the editors and the reviewers. Any product that may be evaluated in this article, or claim that may be made by its manufacturer, is not guaranteed or endorsed by the publisher.
